# A Support Vector Machine Model Predicting the Risk of Duodenal Cancer in Patients with Familial Adenomatous Polyposis at the Transcript Levels

**DOI:** 10.1155/2020/5807295

**Published:** 2020-06-16

**Authors:** Weiqing Liu, Jian Dong, Shumin Ma, Lei Liang, Jun Yang

**Affiliations:** ^1^Department of Internal Medicine Oncology, First Affiliated Hospital of Kunming Medical University, Kunming, China; ^2^Department of Internal Medicine Oncology, Third Affiliated Hospital of Kunming Medical University, Kunming, China; ^3^Department of Oncology, First Affiliated Hospital of Kunming Medical University, Kunming, China

## Abstract

**Objective:**

Familial adenomatous polyposis (FAP) is one major type of inherited duodenal cancer. The estimate of duodenal cancer risk in patients with FAP is critical for selecting the optimal treatment strategy.

**Methods:**

Microarray datasets related with FAP were retrieved from the Gene Expression Omnibus (GEO) database. Differentially expressed genes were identified by FAP vs. normal samples and FAP and duodenal cancer vs. normal samples. Furthermore, functional enrichment analyses of these differentially expressed genes were performed. A support vector machine (SVM) was performed to train and validate cancer risk prediction model.

**Results:**

A total of 196 differentially expressed genes were identified between FAP compared with normal samples. 177 similarly expressed genes were identified both in FAP and duodenal cancer, which were mainly enriched in pathways in cancer and metabolic-related pathway, indicating that these genes in patients with FAP could contribute to duodenal cancer. Among them, Cyclin D1, SDF-1, AXIN, and TCF were significantly upregulated in FAP tissues using qRT-PCR. Based on the 177 genes, an SVM model was constructed for prediction of the risk of cancer in patients with FAP. After validation, the model can accurately distinguish FAP patients with high risk from those with low risk for duodenal cancer.

**Conclusion:**

This study proposed a cancer risk prediction model based on an SVM at the transcript levels.

## 1. Introduction

FAP is an autosomal dominant inherited syndrome manifested as a mass of adenomatous colorectal polyps caused by APC gene mutations, which almost inevitably develops into duodenal cancer at an average age of 35 to 40 years [1]. Duodenal cancer has become the second leading cause of death in patients with the disease [2]. Family identification and subsequent screening programs have significantly reduced morbidity and mortality in duodenal cancer. As a precancerous lesion, colectomy remains the best preventive treatment [3, 4]. However, the appropriate timing of surgery and which endoscopic findings indicate surgery still remain challenging [5]. Thus, it is necessary to estimate duodenal cancer risk in patients with FAP through endoscopic surveillance procedures [6]. The Spigelman scoring system has been used to stratify malignant tumors of FAP patients based on the size, morphology, number, and dysplasia of duodenal polyps under endoscopy [7]. However, increasing evidence suggests that the Spigelman scoring system underestimates the risk of duodenal cancer in patients with FAP along with duodenal polyposis [8, 9]. Therefore, it is necessary to develop new models to predict the risk of cancer in patient with FAP. Because FAP is a genetic disease, surgical treatment after the disease essentially cannot eliminate the risk of recurrence of the disease in patients and has a very high risk of carcinogenesis. In addition, gene mutations associated with FAP are continuously discovered as research into the disease progresses, suggesting that there is a genetic background difference in patients with FAP. Due to the combined effects of the patient's living environment, diet structure, age, and sex, etc., the etiology of FAP is complicated, and there are many uncertainties in treatment and rehabilitation. This requires us to be able to analyze the characteristics of FAP on the basis of differences in genetic background and other factors, to provide recommendations for rehabilitation prognosis, and to guide the choice of treatment methods.

SVM is a supervised learning model commonly used in machine learning, proposed by Cortes and Vapnik in 1995 [10]. Early diagnosis and prognosis of cancer have become a necessary condition for cancer research because they can promote subsequent clinical management of patients. Machine learning tools detect critical features from complex data sets. Among them, SVM has been widely used in cancer research to develop predictive models, resulting in effective and predictable models [11–13]. For example, recent study performed machine learning analysis of DNA methylation profiles to distinguish primary lung squamous cell carcinomas from head and neck metastases [14]. Another study identified characteristic genes associated with vascular invasion in hepatocellular carcinoma, which was validated by SVM [15]. The GEO, an online public database provided by the NCBI in 2000, has been one of the most comprehensive gene expression databases. Based on this database, we comprehensively analyzed gene expression pattern related with FAP and duodenal cancer at the transcript levels. The similarly expressed genes between FAP and duodenal cancer were identified, which were differentially expressed compared to normal cases. Moreover, we constructed a cancer risk prediction model in patients with FAP based on an SVM at the transcript levels.

## 2. Materials and Methods

### 2.1. Microarray Dataset Preparation

The microarray data related to FAP were retrieved from the GEO (http://www.ncbi.nlm.nih.gov/geo/) including GSE111156 [16] and GSE65270 datasets [17]. Corresponding clinical data were also obtained from the GEO database. The gene expression data of GSE111156 and GSE65270 datasets were generated by Affymetrix Human Transcriptome Array 2.0 or Affymetrix Human Gene 1.0 ST Array platform, respectively. The GSE111156 dataset included 24 FAP cases, 12 normal cases, and 12 adenocarcinoma cases. Furthermore, there were 40 FAP cases in the GSE65270 dataset. The GSE111156 dataset was used as a training set, and the GSE65270 dataset was used as a validation set. The expression data were analyzed by Z-score transformation using Linear Models for Microarray data (limma) package (version 3.34.7; https://bioconductor.org) in R3.4.1 [18].

### 2.2. Microarray Data Processing

Based on the annotation information of the GPL17586 platform, the microarray raw CEL files were annotated into the gene expression data, thereby constructing a gene expression matrix. Since an individual gene may have multiple expression data, based on the gene expression matrix, the repeated expression data of each gene were removed, leaving only the maximum expression of the gene. The similarity test of the samples was performed on the expression matrix, and it was preliminarily determined whether there was a difference in the similarity between the samples at the transcript level.

### 2.3. Analysis of Differentially Expressed Genes between FAP and Normal Samples

First, based on the gene expression matrix, we constructed a grouping matrix. The grouping matrix contained the grouping information of the samples, which provided the grouping information for differential expression analysis. Second, a difference comparison matrix was constructed, which specified a pair of samples to compare with each other for downstream analysis. Herein, we specified a comparison between normal samples and FAP samples. Differential expression analysis was performed between 24 cases of FAP and 12 cases of normal samples using limma package (version 3.34.7) in R3.4.1. The adjusted *P* value ≤ 0.05 was set as the cutoff criterion.

### 2.4. Analysis of Similarly Expressed Genes between FAP and Adenocarcinoma Samples

Similar to the identification of differentially expressed genes between FAP and normal samples, we constructed a grouping matrix based on the gene expression matrix using 24 FAP cases, 12 normal cases, and 12 adenocarcinoma cases. The grouping matrix included the grouping information of the samples and provided grouping information for differential expression analysis: the normal samples and disease samples (including 24 FAP and 12 adenocarcinoma samples). The difference between FAP and adenocarcinoma was smaller than that between normal samples and disease samples. Such differences can be used to characterize similar expression characteristics between FAP and adenocarcinoma. Then, a difference comparison matrix was constructed. The difference comparison matrix specified the pair of samples to be compared with each other for downstream analysis. A comparison between normal samples and disease samples (FAP and adenocarcinoma) was specified. Next, we used the R language limma package to detect differentially expressed genes. The adjusted *P* value ≤ 0.05 was set as the filter condition. Ultimately, differentially expressed genes were identified between normal and disease samples.

### 2.5. Functional Enrichment Analysis

After obtaining differentially expressed genes, gene set enrichment analysis (GSEA; http://software.broadinstitute.org/gsea/index.jsp) was performed to identify GO processes [19]. A *P* value ≤ 0.01, *q* − value ≤ 0.01, and Jaccard degree > 0.375 were used as thresholds. After that, similar gene functions were annotated based on the GO database. A Kyoto Encyclopedia of Genes and Genomes (KEGG) pathway enrichment analysis of these differentially expressed genes was carried out using the Database for Annotation, Visualization and Integrated Discovery (DAVID) version 6.8 (https://david.ncifcrf.gov/) [20, 21]. A *P* value < 0.05 was considered to be significantly enriched.

### 2.6. SVM Classifier Construction

SVMs are commonly used to supervise learning, which are primarily used for classification and regression. Since this study was designed to compare FAP-normal, FAP-adenocarcinoma, adenocarcinoma-normal, the GSE111156 dataset was used as a training set. Sigmoid was used to select the SVM model. We performed an examination to select the optimal kernel. The differentially expressed genes were used to construct recursive feature elimination (RFE) analysis [22], which could be used to screen the optimal feature genes in the training dataset. The optimal feature genes were subsequently utilized to construct the SVM classifier [15]. The GSE65270 data was set as a verification set to validate the classifier model and evaluate the risk of cancerization in FAP cases.

(1) $$\begin{array}{c} k\left(x,x^{`}\right)={\left(\overset{n}{\underset{k=1}{\sum }}\exp\left(-\sigma {\left(x^{k}-{x^{`}}^{k}\right)}^{2}\right)\right.}^{d}, \end{array}$$

where *x*^*k*^ is the *k*^th^ component of *x*.

### 2.7. Quantitative Real-Time PCR (qRT-PCR)

Total RNA was extracted from 12 pairs of FAP tissues and normal tissues using TRIzol reagent (Invitrogen), which was reverse transcribed into cDNA. qRT-PCR was carried out on the ABI PRISM 7500 Real-Time PCR System (Applied Biosystems, Foster City, CA). Primers of Cyclin D1, SDF-1, AXIN, and TCF are listed in Table 1. GAPDH was used as an internal control. The relative expression levels of mRNAs were calculated with the 2^−*ΔΔ*CT^ method. All experiments were repeated at least three times. Our study was approved by the ethics committee of the First Affiliated Hospital of Kunming Medical University. All patients provided written informed consent.

## 3. Results

### 3.1. Sample Similarity Test

**Figure 1:**
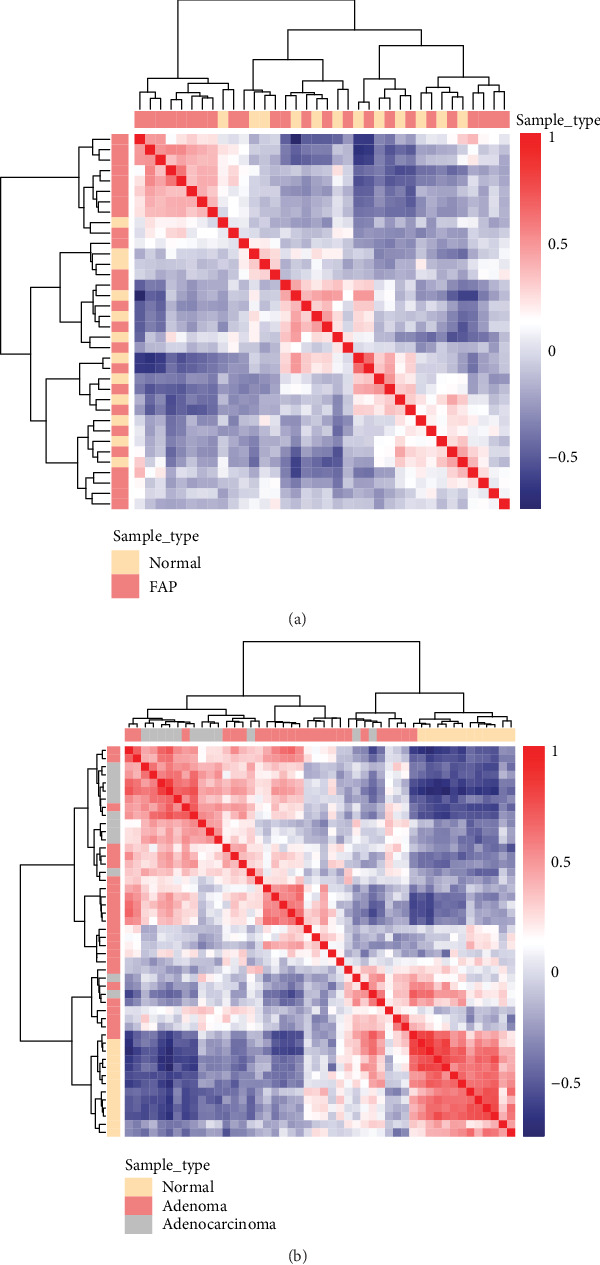
Sample clustering at the transcription level. Sample similarity test was performed using the GSE111156 dataset. (a) FAP vs. normal. (b) FAP vs. normal, FAP vs. adenocarcinoma, or normal vs. adenocarcinoma. At the top of the heat map, sample type is shown. Red represents positive correlation, and blue represents negative correlation. FAP: familial adenomatous polyposis.

Using the gene expression matrix, the correlation coefficient matrix between samples in the GSE111156 dataset was obtained, followed by the Euclidean distance of the correlation coefficient between the samples. As shown in the heat map, we found the differences in transcription levels between normal samples and FAP or adenocarcinoma cases (Figures 1(a) and 1(b)). Compared to the difference between normal samples and FAP or adenocarcinoma cases, the difference between FAP and adenocarcinoma cases was smaller. Therefore, we performed further downstream analysis.

### 3.2. Identification of Differentially Expressed Genes in FAP Compared with Normal Samples

**Figure 2:**
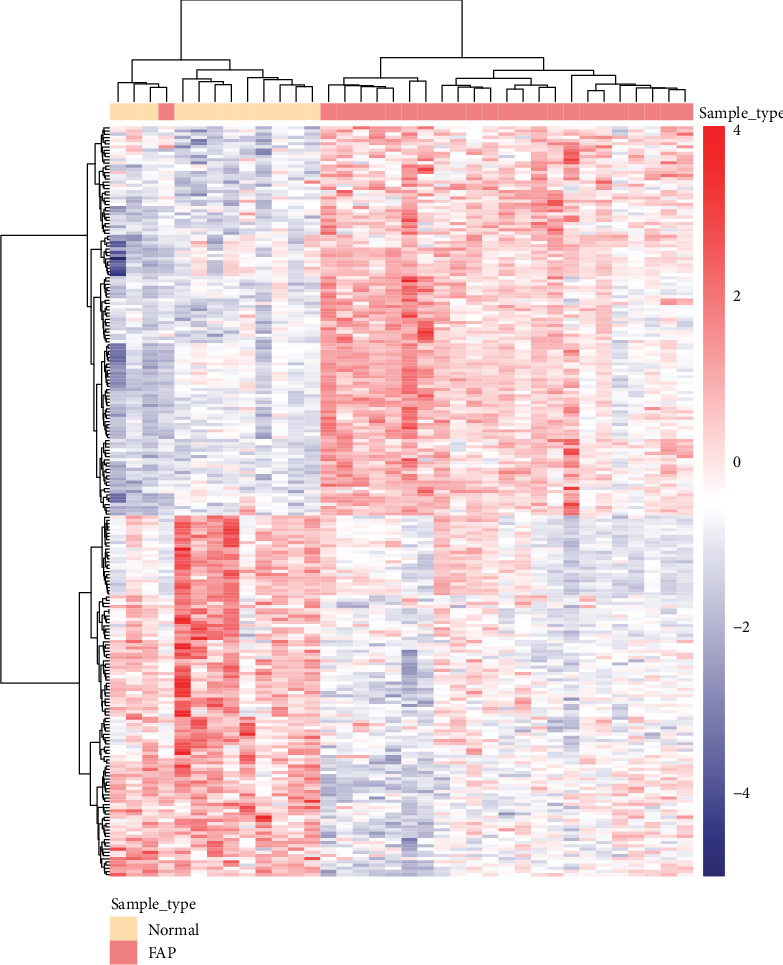
Differential expression gene clustering using heat map. The differentially expressed genes between normal samples and FAP samples were used to construct expression matrices, and *z*-score was used for data standardization. The samples and genes were clustered by the Euclidean distance. At the top of the heat map, sample type is shown. Red represents upregulated, and blue represents downregulated genes in FAP compared to normal samples. FAP: familial adenomatous polyposis.

Herein, we specified a comparison between normal samples and FAP samples. The differential expression analysis was performed using the R language package limma. Among the results obtained, the corrected *P* value ≤ 0.05 was set as the filter condition. Finally, we identified 196 differentially expressed genes in FAP compared with normal tissues (Supplementary Table 1). In Figure 2, the difference in patterns of differentially expressed genes between FAP and normal samples is shown.

### 3.3. Functional Enrichment Analysis of Differentially Expressed Genes in FAP Compared with Normal Samples

**Figure 3:**
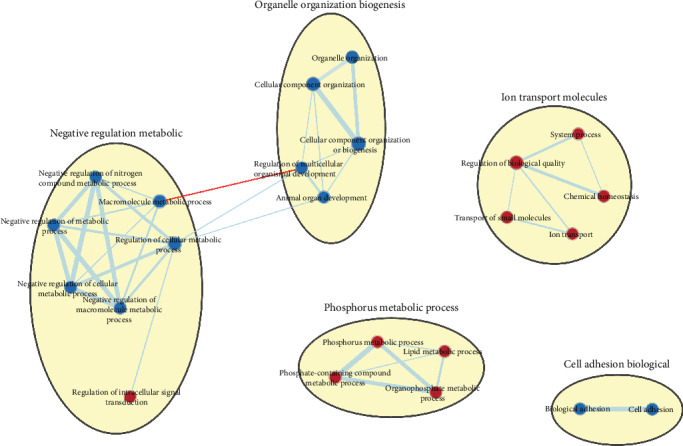
Gene enrichment and functional annotations of differentially expressed genes in FAP compared with normal tissues. Blue represents the highly expressed genes in FAP, and red represents the highly expressed genes in normal tissues.

After obtaining differentially expressed genes between normal samples and FAP samples, to elucidate the function of differentially expressed genes, GSEA software was used to perform functional enrichment analysis enriched by differentially expressed genes. *P* value = 0.01, *q* − value = 0.01, and Jaccard degree > 0.375 were set as thresholds. Moreover, similar gene functions were annotated based on the GO database. We found that, among the differentially expressed genes, the genes that were highly expressed in FAP were extensively enriched into negative regulation metabolic, organelle organization biogenesis, and cell adhesion biological processes. The genes that were highly expressed in normal samples were mainly enriched in the function of phosphorus metabolic process and ion transport molecules (Figure 3).

**Figure 4:**
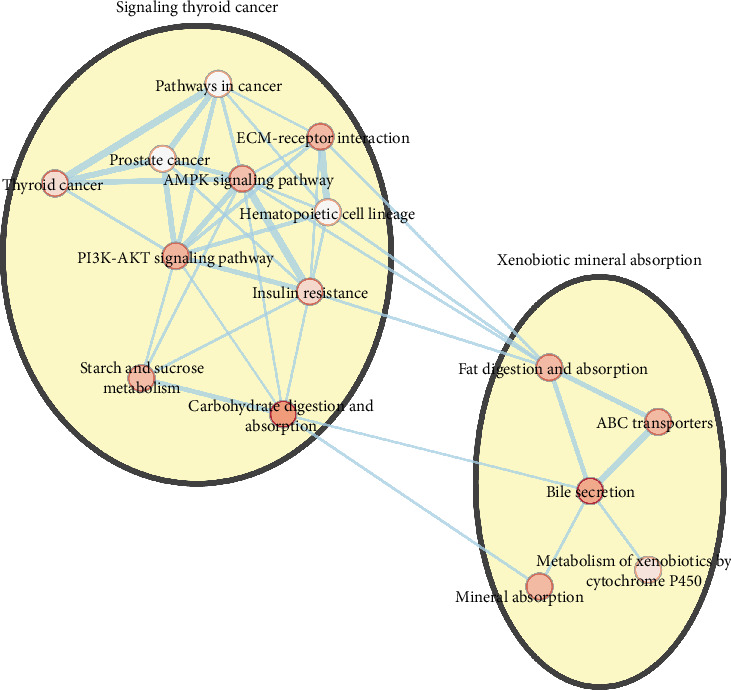
KEGG pathway enrichment analysis of differentially expressed genes in FAP compared with normal tissues. The circle represents the signal pathways. The color in the circle represents the *P* value. The darker the color, the smaller the *P* value.

**Figure 5:**
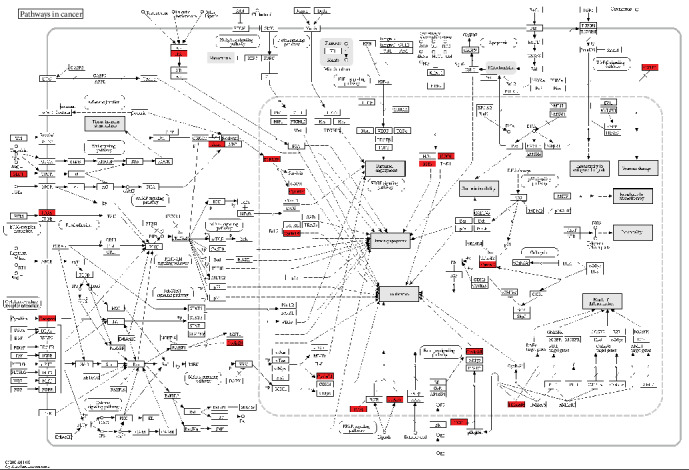
Pathways in cancer enriched by differentially expressed genes in FAP compared with normal tissues. Green suggests that the genes are highly expressed in FAP compared to normal tissues, and red suggests that the genes are lowly expressed in FAP compared to normal tissues.

Furthermore, we performed pathway enrichment analysis to identify functional features of differentially expressed genes using the online KEGG pathway enrichment analysis tool DAVID. We found that a total of 191 differentially expressed genes were enriched in 15 KEGG pathways. Among them, genes that were highly expressed in FAP were enriched in many pathways such as signaling thyroid cancer and xenobiotic mineral absorption (Figure 4), particularly in pathways in cancer (Figure 5).

### 3.4. Identification of Similarly Expressed Genes in FAP and Adenocarcinoma

**Figure 6:**
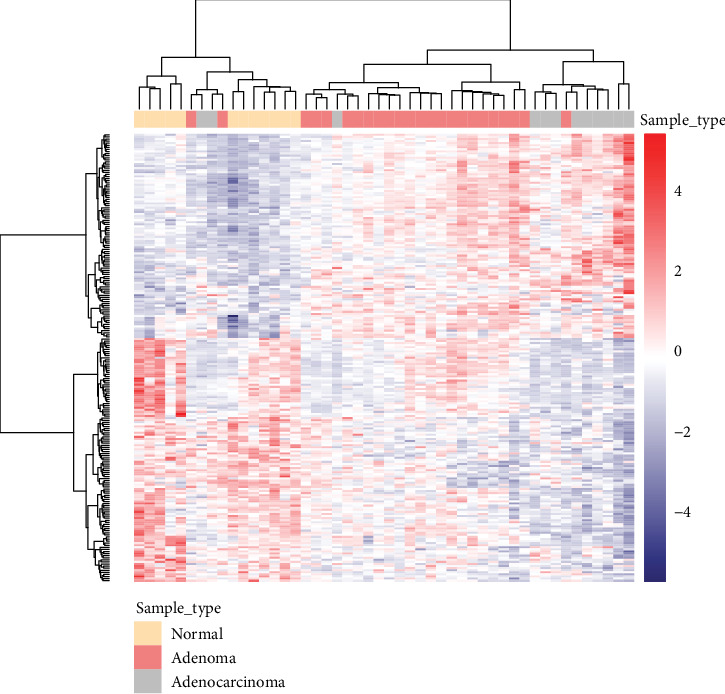
Differential expression gene clustering using heat map. The differentially expressed genes between normal samples and FAP or adenocarcinoma samples were used to construct expression matrices, and *z*-score was used for data standardization. The samples and genes were clustered by the Euclidean distance. At the top of the heat map, sample type is shown.

The differential expression gene detection by comparing FAP or adenocarcinoma samples and normal samples was performed using the R language package limma. The genes with the corrected *P* value ≤ 0.05 were identified as differentially expressed genes. In Figure 6, we identified 177 differentially expressed genes, which could distinguish between normal samples and FAP or adenocarcinoma samples but could not distinguish between FAP and adenocarcinoma.

### 3.5. Functional Enrichment Analysis of Similarly Expressed Genes in FAP and Adenocarcinoma

**Figure 7:**
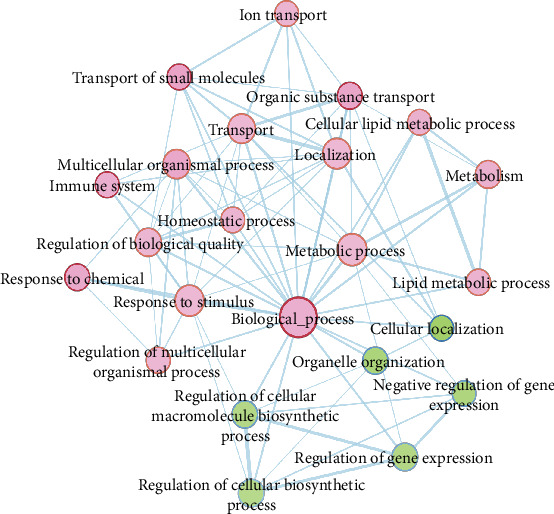
Gene enrichment and functional annotations. Red represents the highly expressed genes in FAP and adenocarcinoma, and green represents the highly expressed genes in normal tissues.

**Figure 8:**
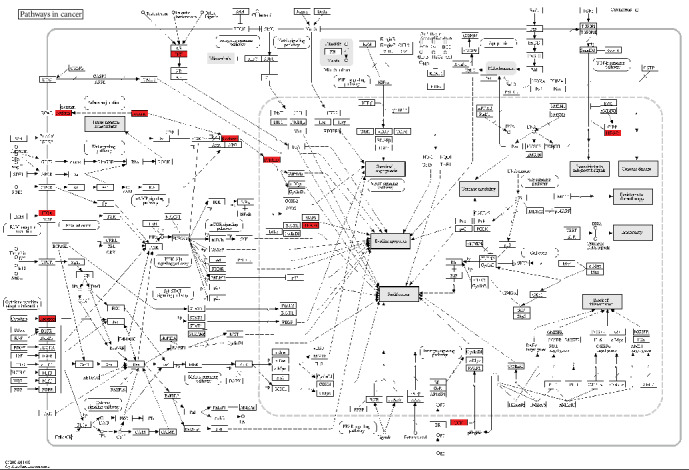
Pathways in cancer enriched by differentially expressed genes in FAP and adenocarcinoma. Green suggests that the genes are highly expressed in FAP and adenocarcinoma, and red suggests that the genes are lowly expressed in FAP.

**Figure 9:**
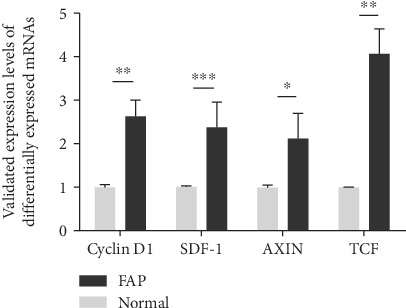
Validation of genes in the cancer-related pathways in FAP tissues, including Cyclin D1, SDF-1, AXIN, and TCF.

After that, enrichment analysis of these differentially expressed genes was performed using GSEA software, with a *P* value = 0.01, *q* − value = 0.01, and Jaccard degree > 0.375 as thresholds. The similar gene functions were annotated based on the GO database. The results showed that among the differentially expressed genes, genes that were highly expressed in FAP and adenocarcinoma were mainly enriched in metabolic processes (Figure 7). These 177 genes reflected the similarity of the expression level both in FAP and adenocarcinoma. We defined these 177 genes as the similarity gene set at the expression level in FAP and adenocarcinoma. Through the pathway enrichment analysis of these genes, we found that these pathways that enriched these genes were significantly associated with cancer-related pathways (Figure 8). Interestingly, there were 65 overlapping genes between the 177 similarity expressed gene sets and the 191 differentially expressed genes in FAP compared to normal samples, which reflected some gene expression changes in FAP at the expression level that tended to be similarly expressed in adenocarcinoma. qRT-PCR results showed that, among genes in the cancer-related pathway, Cyclin D1, SDF-1, AXIN, and TCF were all significantly upregulated in FAP tissues compared to normal tissues (Figure 9), indicating their roles in the development of FAP.

### 3.6. Construction of FAP and Adenocarcinoma Binary Classification Model Based on SVM

Based on the similarity measurement gene set of 177 genes for FAP and adenocarcinoma at the transcript levels, machine learning was used to construct a SVM-based binary model. Using the GSE111156 dataset as a training set, this classification model can distinguish between FAP and adenocarcinoma. After training, the model had a good discrimination degree of FAP and adenocarcinoma. The error rate was 0, and the relevant parameters of the model are shown in Table 2.

**Figure 10:**
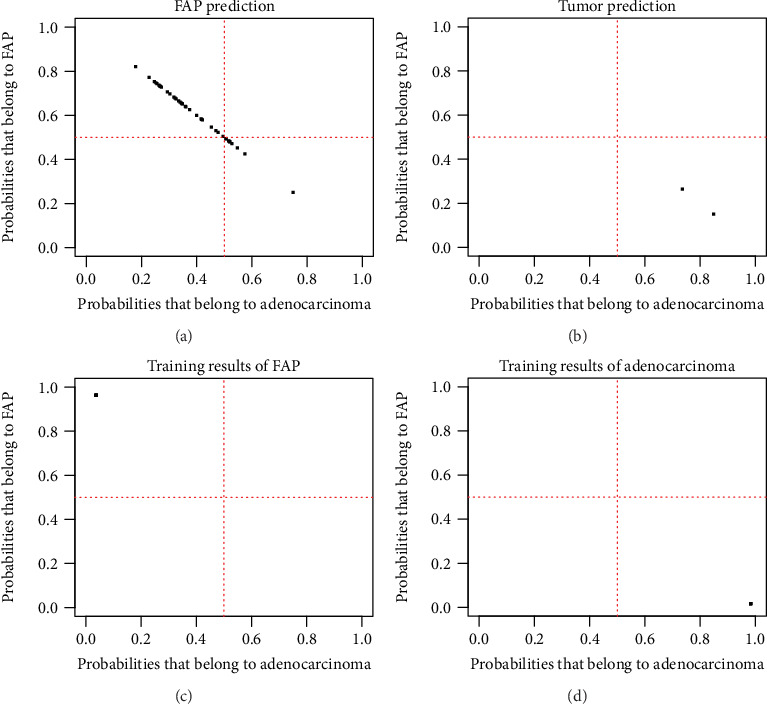
The risk of adenocarcinoma in FAP based on an SVM: (a) FAP prediction; (b) adenocarcinoma prediction; (c) training result of FAP in the GSE65270 dataset; (d) training result of adenocarcinoma in the GSE65270 dataset.

The GSE65270 dataset was used to validate the binary classification model (Figure 10). Using a 50% classification probability as a criterion, we can define the similarity between the 177 genes of any sample in FAP and cancer. If the judgment results showed that the pathological similarity probability to cancer was greater than 50%, the case was considered to be closer to cancer at the transcription levels. Furthermore, these 177 genes showed a high degree of similarity in cancer-related pathways. Therefore, we believed that once the FAP case had a cancer similarity probability greater than 50%, suggesting that the case had a high risk of cancer. According to the model, 6 of the 40 FAP cases in the GSE65270 dataset had a high cancer risk of more than 50%, accounting for 15% of the entire cases.

## 4. Discussion

In this study, we constructed an SVM model that might predict the risk of adenocarcinoma in patients with FAP at the transcript levels.

We identified 196 differentially expressed genes in 24 cases of FAP compared with 12 cases of normal tissues using microarray. Although falling prices and mature technology have made next-generation sequencing technology as the first choice in many ways, the transition from microarray technology to next-generation sequencing technology is a long and iterative process. Microarray technology is easier to operate than next-generation sequencing and does not require complex, intensive labor sample preparation, and massive data analysis. Furthermore, there are many tools available for microarray technology in data analysis, and uniform results are easy to be obtained by using the main methods. Compared with the cost of next-generation sequencing, microarray technology is more economical and cost-effective, especially when processing large-scale samples. Therefore, in this study, microarray data were used to identify differentially expressed genes. Previous study identified 84 differentially expressed genes in FAP compared to the corresponding normal mucosa, which revealed the gene deregulation during adenoma formation [23]. To explore the biological dysregulation under adenoma formation, we performed enrichment analysis of these differentially expressed genes. We found that the highly expressed genes in FAP were mainly enriched into negative regulation metabolic processes and cell adhesion biological processes, which play a critical role in FAP [24, 25]. KEGG pathway enrichment analysis results showed that the genes that were highly expressed in FAP were enriched in several KEGG pathways related with cancer such as signaling thyroid cancer and xenobiotic mineral absorption, particularly in pathways in cancer. Among genes in pathways in cancer, Cyclin D1, SDF-1, AXIN, and TCF were all significantly upregulated in FAP tissues compared to normal tissues. FAP is involved in many extracolonic organs, such as the thyroid [26, 27]. These genes were enriched into thyroid cancer-related pathways, indicating that they could contribute to thyroid cancer in patients with FAP. Furthermore, recent study proposed that differential expression of genes in the Wnt pathway could be considered a potential biomarker for duodenal cancer stratification [28]. Therefore, these differentially expressed genes could be involved in the development of FAP.

After identification of differentially expressed genes in FAP, we also analyzed the gene expression profile by comparing FAP and duodenal cancer with normal samples. 177 genes were differentially expressed in FAP and duodenal cancer compared with normal samples. More importantly, these differentially expressed genes had similar expression pattern in FAP and duodenal cancer. To illuminate potential functions of these genes, functional enrichment analysis was performed. GSEA results showed that highly expressed genes in FAP and duodenal cancer were mainly enriched in metabolic processes, indicating that these genes could be involved in metabolic processes both in FAP and duodenal cancer. According to KEGG pathway enrichment analysis results, these genes were mainly enriched in pathways in cancer. Moreover, we found that 65 genes were differentially expressed in FAP compared with normal samples, which had similar expression pattern in duodenal cancer. This indicated that the expression level of partial gene in FAP tends to be similarly expressed with cancer, and these genes could be significantly associated with cancer. Recent study identified differentially expressed genes by comparing duodenal adenoma vs. carcinoma sequence in FAP transcriptional profiling [16]. Functional enrichment analysis revealed that these genes could be involved in several signaling pathways associated with duodenal cancer.

Based on 177 similarly expressed genes in FAP and duodenal cancer, we constructed an SVM-based binary classification model. Our results showed that the model can accurately distinguish between FAP and duodenal cancer using the GSE111156 dataset. To further validate the model, the GSE65270 dataset was used as a validation set. Our model predicted that 6 of the 40 FAP in the GSE65270 dataset had a cancer similarity probability of more than 50%, which was a high cancer risk pathology, accounting for 15% of the entire FAP cases. Defining the risk of cancer in FAP can guide not only the choice of treatment options but also the recovery of patients after surgery. At the same time, based on the prediction of cancer risk of gene expression level, its operation is simple, and the sample RNA can be obtained by biopsy. A previous study screened 15 genes to predict the risk of colon cancer recurrence based on SVM [29]. A recent study constructed a 19-miRNA SVM classifier for ovarian cancer patients, which may be considered a potential biomarker for ovarian cancer prognosis [30]. Another study established an SVM prediction model for gastric cancer [31]. However, we firstly proposed a cancer risk model for patients with FAP.

The limitations of this study should be noted. First, the sample size with FAP was small. However, we performed qRT-PCR assay to validate the expression of key genes in FAP. Second, although this study was based on GEO related to FAP and was verified by machine learning, prospective studies in different populations should be required to validate our findings.

In summary, we constructed an SVM model that can predict duodenal cancer risk in FAP at the transcript levels, which may help predict individual cancer risk and help clinicians manage patients with FAP.

## 5. Conclusion

In our study, we screened differentially expressed genes of FAP or adenocarcinoma compared with normal tissues and identified similarly expressed genes between FAP and adenocarcinoma. Furthermore, functional enrichment analysis was performed for these differentially expressed genes. By constructing and verifying SVM classifier, characteristic genes were obtained. Furthermore, a risk prediction model was constructed, which could predict the risk of duodenal cancer in patients with FAP. However, the model required further validation.

## Figures and Tables

**Table 1:** Primer information for qRT-PCR.

Genes	Primer sequence
Cyclin D1	5′-CGCTGGAGCCCGTGAAA-3′ (forward)
	5′-GGATGGAGTTGTCGGTGTAGATG-3′ (reverse)
SDF-1	5′-ACGCCAAGGTCGTGGTC-3′ (forward)
	5′-AGCTTCGGGTCAATGCA-3′ (reverse)
AXIN	5′-AGCCCTCCCACCTCTTCATC-3′ (forward)
	5′-ACCTTCCTCTGCGATCTTGTCT-3′ (reverse)
TCF	5′-ACCCAGCCTACACCACCCT-3′ (forward)
	5′-GTCTTTGTCCACCACGCACT-3′ (reverse)

**Table 2:** The parameters of the SVM model for FAP.

Support vector machine object of class “ksvm”
SV type: C-bsvc (classification)
Parameter: cost *C* = 100
ANOVA RBF kernel function
Hyperparameter: sigma = 10 degree = 1
Number of support vectors: 19
Objective function value: -0.0807
Training error: 0
Probability model included

## Data Availability

The (data type) data used to support the findings of this study are included within the supplementary information file(s).

## Abbreviations

FAP: familial adenomatous polyposis
SVM: support vector machine
GEO: gene Expression Omnibus
GSEA: gene set enrichment analysis
KEGG: kyoto Encyclopedia of Genes and Genomes
DAVID: database for Annotation, Visualization and Integrated Discovery
limma: linear Models for Microarray data
RFE: recursive feature elimination.

## Supplementary Materials

Supplementary Materials Supplementary Table 1: 196 differentially expressed genes in FAP compared with normal tissues.
